# Effect of Strain Rate and Temperature on the Tensile Properties of Long Glass Fiber-Reinforced Polypropylene Composites

**DOI:** 10.3390/polym15153260

**Published:** 2023-07-31

**Authors:** Qiaoyu Wang, Jianbin Wang, Anheng Wang, Chaoqun Zhou, Jiale Hu, Fei Pan

**Affiliations:** 1 School of Mechanical Engineering, Anhui Polytechnic University, Wuhu 241000, China; wangqiaoqu@163.com (Q.W.); wahazf@ahpu.edu.cn (A.W.); 15056740996@163.com (J.H.); panfei7410@163.com (F.P.); 2 Wuhu Hengxin Auto Interior Manufacturing Corporation Limited, Wuhu 241009, China; zhouchaoqun@anhuihx.com

**Keywords:** LGFRP, tensile strength, tensile fracture stress, fracture morphology

## Abstract

Strain rate and temperature are influential factors that significantly impact the mechanical properties of long glass fiber-reinforced polypropylene composites. This study aims to investigate the tensile properties of these composites, analyzing the effects of temperature, strain rate, and their interplay on variables such as tensile stress, tensile strength, fracture stress, and fracture morphology through a series of comprehensive tensile experiments. The experimental results demonstrate a notable increase in both tensile strength and tensile fracture stress when the temperature is set at 25 °C, accompanied by strain rates of 10^−4^, 10^−3^, 10^−2^, and 10^−1^ s^−1^. Conversely, a significant decrease is observed in the aforementioned properties when the strain rate is fixed at 10^−4^, while varying temperatures of −25 °C, 0 °C, 25 °C, 50 °C, and 75 °C are applied. At lower temperatures, cracks manifest on the fracture surface, while matrix softening occurs at higher temperatures. Additionally, in the context of strain rate–temperature coupling, the decreasing trend of both tensile strength and tensile fracture stress decelerates as the temperature ranges from −25 °C to 75 °C at a strain rate of 10^−1^, compared to 10^−4^ s^−1^. These findings highlight the significant influence of both strain rate and temperature on high fiber content long glass fiber-reinforced polypropylene composites.

## 1. Introduction

Long glass fiber-reinforced polypropylene composite is a lightweight composite material composed of glass fibers and a polypropylene matrix. This material offers numerous advantages, including low density, high strength, specific stiffness, excellent impact and deformation resistance, dimensional stability, durability, and favorable performance for recycling. As a result, it finds widespread use in aerospace, automotive, and other industries [1,2,3,4]. Particularly in the automotive sector, the utilization of glass fiber-reinforced polypropylene composites in components such as car body door panels, front frames, bumper brackets, and interior decorations enables weight reduction and the substitution of steel with plastic [5,6,7,8,9,10,11]. During driving or in the event of an accident, automotive parts are subjected to varying strain rate environments, and ambient temperatures can vary across regions. Long glass fiber-reinforced polypropylene may exhibit different properties in low-temperature environments. It is necessary to investigate the tensile properties at low temperatures to ensure the reliability of the design at low temperature environments and to ensure that the material is not subjected to sudden failures when stressed. Therefore, the changes in mechanical properties resulting from strain rate and temperature have a significant impact on vehicle driving safety.

In recent years, there has been a growing body of research focusing on the mechanical properties of glass fiber reinforced polypropylene. Notably, scholars such as Duan et al. have conducted studies on the tensile properties of long glass fiber-reinforced polypropylene composites produced using air laying web technology with varying fiber contents of 50% and 20% [5,12,13]. It was found that within the strain rate range of 0.001 s^−1^ to 50 s^−1^, the ultimate tensile strength and ultimate strain exhibited significant improvements, with more pronounced effects observed at the fiber content of 50%. Cui et al. [14] investigated the tensile properties and damage modes of long glass fiber reinforced polypropylene composites manufactured through injection molding at low, medium, and high strain rates (ranging from 0.001 s^−1^ to 400 s^−1^) with a fiber content of 40%. The study concluded that the ultimate strength and total elongation of LGFPPs increased two-fold and one-fold, respectively. Additionally, the interfacial bonding properties were enhanced, leading to an increase in fracture fiber occurrence and a reduction in fiber drawability. Lu et al. [15] and others fabricated GF/PP composite samples with fiber contents of 0%, 10%, 20%, and 35% using laminated hot-pressing, followed by tensile property testing at strain rates of 10^−4^, 5 × 10^−4^, 2.5 × 10^−4^, and 1.25 × 10^−4^ s^−1^. The test results demonstrated that both the occurrence of tensile fracture and the magnitude of tensile stress increased with higher strain rates. Additionally, researchers such as Kim et al. [16], Schoßig et al. [17], and Zhai et al. [18] have similarly examined the tensile properties of glass fiber-reinforced polypropylene composites at various strain rates.

Zhang et al. [19] evaluated the influence of strain rate on the tensile mechanical properties of glass fiber-reinforced polypropylene composites. Qin et al. [20] and colleagues conducted a comprehensive tensile test on long wave fiber-reinforced polypropylene at various temperatures. The results clearly indicate a significant decrease in tensile strength with increasing temperature. Reis et al. [21] and co-authors investigated the tensile properties of glass fiber reinforced polymers (GFRP) with fiber contents reaching 70% across different temperature settings (20/40/60/80 °C) and strain rates (6 × 10^−5^/1.6 × 10^−4^/1.6 × 10^−3^ s^−1^). The test outcomes reveal that both temperature and strain rate exert considerable influence on the tensile properties of GFRP. Specifically, the ultimate tensile strength is predominantly affected by the strain rate, while the modulus is primarily influenced by temperature. Yu et al. [22] focused on studying the compressive mechanical properties of polypropylene and short glass fiber-reinforced polypropylene with a 25% fiber content under varying loading rates and temperatures. Bajracharya et al. [23,24] investigated the tensile properties of injection molded glass fiber-reinforced mixed plastics under normal and high environmental conditions. The investigation demonstrates that the dynamic mechanical properties of these two materials are sensitive to different strain rates and temperatures. Increasing the loading rate or decreasing the temperature leads to an elevation in modulus and fracture strength, while reducing the elongation at break.

Glass fiber and polypropylene composites are commonly manufactured through processes such as injection molding, hot compaction, or molding [25]. Currently, there is a significant body of research examining the mechanical properties of glass fiber-reinforced composites employing hot pressing and diverse lay-up designs. However, there remains a dearth of studies investigating the tensile mechanical properties of injection molded long glass fiber-reinforced polypropylene under conditions involving temperature and strain rate–temperature coupling. Furthermore, Gonçalves [26] and Li [27] found that long glass fiber-reinforced polypropylene has strong commercial value and development potential, and long glass fiber-reinforced polypropylene with high fiber content finds extensive practical application. Nevertheless, there is limited research available concerning the tensile mechanical properties of long glass fiber-reinforced polypropylene composites containing 60% fiber content under varying strain rates, temperatures, and strain rate–low temperature coupling. Given these research gaps, this article aims to address these issues. Specifically, the study conducts tensile experiments on injection molded long glass fiber-reinforced polypropylene composites with a fiber content of 60% to examine the effects of different strain rates (ranging from 10^−4^ to 10^−1^ s^−1^), temperatures (ranging from −25 to 75 °C), and strain rate–temperature coupling. Additionally, this article employs microscopy to observe the micro-morphology of tensile fractures under different strain rates and temperatures.

## 2. Experimental Design and Characterization

### 2.1. Sample Preparation

**Figure 1:**
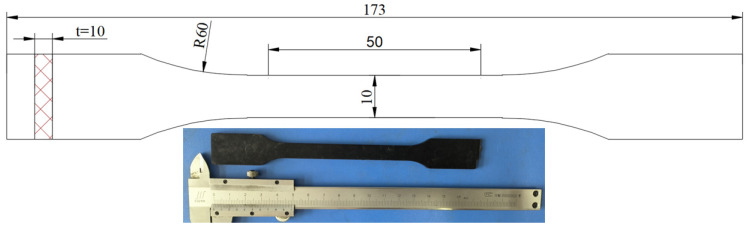
Size figure and tensile entity diagram of LGFPP tensile specimen.

For this experiment, the raw materials used to produce long glass fiber-reinforced polypropylene were polypropylene and glass fiber, both sourced from Wuhu Hengxin Auto Interior Manufacturing Corporation Limited. The material properties of these components are presented in Table 1, with the long glass fiber constituting 60% of the total weight of the long glass fiber-reinforced polypropylene composites. Dumbbell-shaped tensile samples were obtained via injection molding, as depicted in Figure 1. The samples possess a thickness of 4 mm and a width of 10 mm.

### 2.2. Tensile Test Design

**Figure 2:**
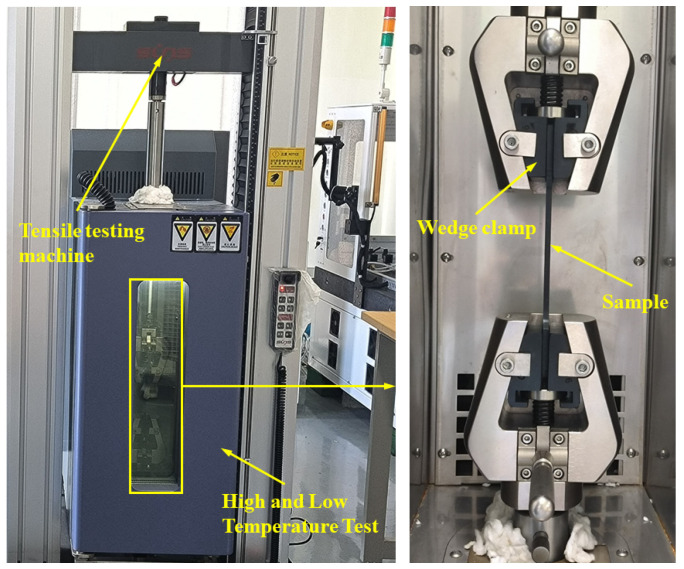
The devices for tensile tests.

The tensile tests conducted in this experiment involved uniaxial static load testing. These tests were performed using an electronic universal testing machine (UTM4104, Shenzhen Suns Technology Stock Co., Ltd., Shenzhen, China) with a maximum load capacity of 10 KN. To prevent sample slippage and ensure secure clamping during the tension process, a wedge-shaped fixture was utilized, as illustrated in Figure 2.

Table 2 presents the test parameter design. Typically, determining the sensitivity of strain rates in tensile tests requires the inclusion of three or more strain rates [18]. Therefore, in this study, tensile tests were conducted at various strain rates: 10^−4^, 10^−3^, 10^−2^, and 10^−1^ s^−1^, at room temperature (25 °C). To examine the influence of ambient temperature on long glass fiber-reinforced polypropylene, a high- and low-temperature test chamber (WGDY-4150S, Shenzhen Suns Technology Stock Co., Ltd., Shenzhen, China) was utilized. The test chamber was equipped with a fan to ensure temperature uniformity, and its adjustable range spanned from −40 °C to 150 °C. Tensile tests were performed at temperatures of −25 °C, 0 °C, 25 °C, 50 °C, and 75 °C using a strain rate of 10^−4^ s^−1^. Prior to the temperature tests, the samples were securely clamped and positioned in the middle of the high and low temperature chamber. The temperature was then adjusted within the chamber to the desired set temperature, and once reached, the tensile tests were conducted. Additionally, to investigate the combined effect of strain rate and temperature, tensile tests were performed at −25 °C, 0 °C, 25 °C, 50 °C, and 75 °C using a strain rate of 10^−1^ and 10^−4^ s^−1^.

### 2.3. Characterization of Experimental Results

The engineering stress–strain curve obtained from the test does not accurately reflect the true stress–strain relationship of the material. Hence, the true stress (*σ*) and true strain (*ε*) are calculated using the Formulas (1) and (2) [28,29,30]:

(1) $$\sigma_{t}=\sigma_{e}\left(1+\varepsilon_{e}\right)$$

(2) $$\varepsilon_{t}=\ln\left(1+\varepsilon_{e}\right)$$

where $\sigma_{t}$ and $\sigma_{e}$ represent the true stress and engineering stress, respectively. $\varepsilon_{t}$ and $\varepsilon_{e}$ denote the true strain and strain engineering strain, respectively.

Tensile fracture strain is a crucial parameter for evaluating material toughness and its ability to absorb impact loads. Greater tensile fracture strain signifies improved toughness and the capacity to absorb more energy during the tensile process [31]. The tensile fracture strain of the material is calculated by the Formula (3) [32].

(3) $$\varepsilon =\frac{l-l_{0}}{l_{0}}\times 100\%$$

where *l* and $l_{0}$ represent the tensile length and the initial length of the sample, respectively.

In order to analyze the characteristics and effects of different strain rates and temperatures on the fracture surface, the fractured specimens were observed using a scanning electron microscope (S-4800, Hitachi, Ltd., Tokyo, Japan) and an ultra-depth of field microscope (VHX-5000, KEYENCE, Tokyo, Japan).

## 3. Experimental Results and Analysis

### 3.1. The Effect of Strain Rate on LGFPP

**Figure 3:**
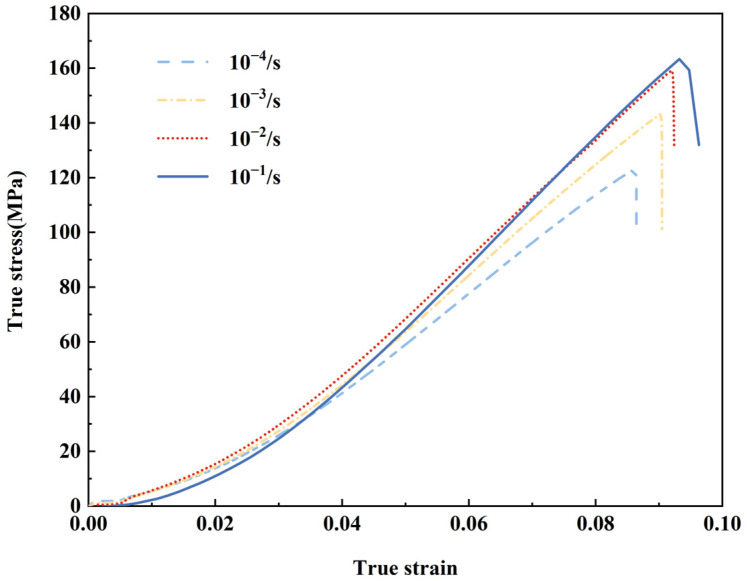
True stress–strain curves for LGFPP at different strain rates.

The tensile test of long glass fiber-reinforced polypropylene was conducted at four different strain rates under an ambient temperature of 25 °C. The occurrence of a loud sound accompanied the tensile fractures, and the corresponding fracture times were measured as follows: 902.3 s, 94.7 s, 8.6 s, and 1.1 s, respectively. These results indicate a decrease in the tensile fracture time with an increase in strain rate. Figure 3 illustrates the real stress–strain curve, revealing an ascending and descending pattern characterized by increasing maximum strain and ultimate strength with higher strain rates. The curves primarily exhibit a rising section, wherein the sample experiences rapid breakdown after reaching maximum stress. The relationship within the rising section before reaching maximum stress is nonlinear. The reason for the tendency of the curve to be nearly linear in the latter part of the upward section may be that the long glass fibers bear the stress and are able to maintain a relatively stable load. Notably, at a high strain rate, the sample undergoes rapid failure without sufficient time to distribute the load. Consequently, the stress–strain curve at a strain rate of 10^−1^ s^−1^ intersects with the other three curves during the ascending section. The calculated tensile fracture strain, determined using Formula (3), exhibits a 12.0% increase as the strain rate rises from 10^−4^ to 10^−1^ s^−1^.

**Figure 4:**
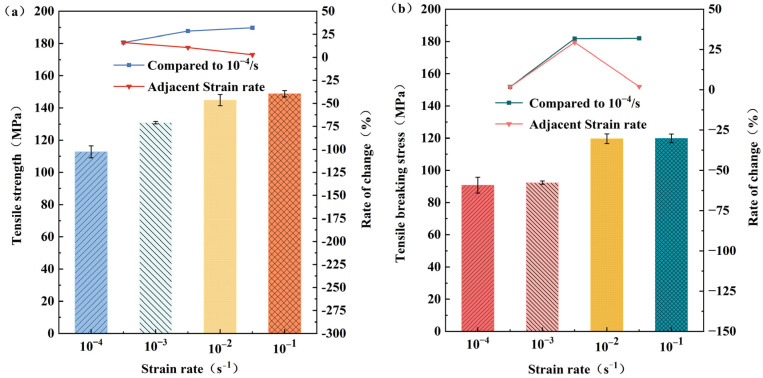
Variation curves of tensile strength and tensile fracture stress of long glass fiber-reinforced polypropylene at different strain rates: (**a**) Diagram of tensile strength variation; (**b**) diagram of tensile breaking stress variation.

Figure 4a illustrates the variation in tensile strength at different strain rates. It is observed that the tensile strength exhibits a continuous and noticeable increase as the strain rate rises, although the rate of growth gradually diminishes. As noted by Schoßig et al. [17] and Shokrieh et al. [33], the enhancement in tensile strength can be attributed to the shortened pull-off time caused by the higher strain rate. This limited time prevents the development and rupture of fiber and matrix defects, as well as the breakage of intra-molecular chemical bonds, thereby augmenting the intermolecular forces. However, the deceleration in the growth trend may be attributed to the intensified strain rate, resulting in fiber breakage at various points within the specimen. Consequently, the intermolecular forces are diminished.

Figure 4b illustrates the variation in tensile fracture stress at different strain rates. In comparison to a strain rate of 10^−4^ s^−1^, the tensile fracture stress increases by 2.2%, 31.8%, and 32.0% at a strain rate of 10^−1^ s^−1^. Additionally, the adjacent sides exhibit increases of 2.2%, 29.5%, and 0.2% in tensile fracture stress, respectively. These findings indicate that the tensile fracture stress of long glass fiber-reinforced polypropylene rises with higher strain rates, particularly in the low strain rate range of 10^−4^ to 10^−2^ s^−1^. Consequently, the strain rate significantly impacts the tensile properties of long glass fiber-reinforced polypropylene composites. 

The tensile fracture stress exhibits minimal variation when the strain rate ranges from 10^−2^ to 10^−1^ s^−1^. This observation can be attributed to the inherent weakness of the polypropylene matrix during the tensile process. Initially, the high load causes the glass fibers to break, while the surrounding intact long glass fibers absorb the energy transmitted from the fractured ones, leading to a stress concentration phenomenon. Subsequently, the unbroken long glass fibers, bearing excessive loads, experience brittle fracture, ultimately resulting in sample failure. Notably, when the long glass fiber content reaches 60%, the stress concentration arising from the excessive loading of long glass fibers becomes more pronounced. Moreover, at higher strain rates, the long glass fibers fracture independently and at multiple locations. This multifaceted fracture pattern allows the long glass fibers to absorb energy from multiple sources, leading to a more rapid and violent specimen fracture, accompanied by more severe damage to the fracture site. Consequently, the fracture time is significantly reduced, while the change in fracture tensile stress becomes negligible within the strain rate range of 10^−2^ to 10^−1^ s^−1^.

### 3.2. The Effect of Temperature on LGFPP

A strain rate of 10^−4^ s^−1^ was selected to facilitate the examination of temperature dependency and minimize the influence of strain rate during the initial stage of the tensile experiments. To gain a more intuitive understanding of the sample’s response to temperature, the temperature was set at intervals of 25 °C, ranging from −25 °C to 75 °C. Notably, the samples exhibited audible fracture accompanied by breaking times of 963, 903, 1017.6, and 1034.4 s, respectively. It is evident that the tensile fracture time increases with each 25 °C increment or decrement in comparison to the reference temperature of 25 °C. Moreover, this increase is particularly pronounced in high-temperature environments. Conversely, in low-temperature environments, the sample undergoes freezing, leading to sluggish movement among the polypropylene matrix molecules. Thus, the tensile fracture time at the same strain rate exceeds that observed at room temperature. As the temperature rises, the movement between the polypropylene matrix and long glass fiber molecules becomes more vigorous. Concurrently, the matrix experiences a softening effect, which becomes increasingly prominent with temperature elevation. Owing to this matrix softening, the stretching process requires more time at higher temperatures, contrasting with the shorter times observed at lower temperatures.

**Figure 5:**
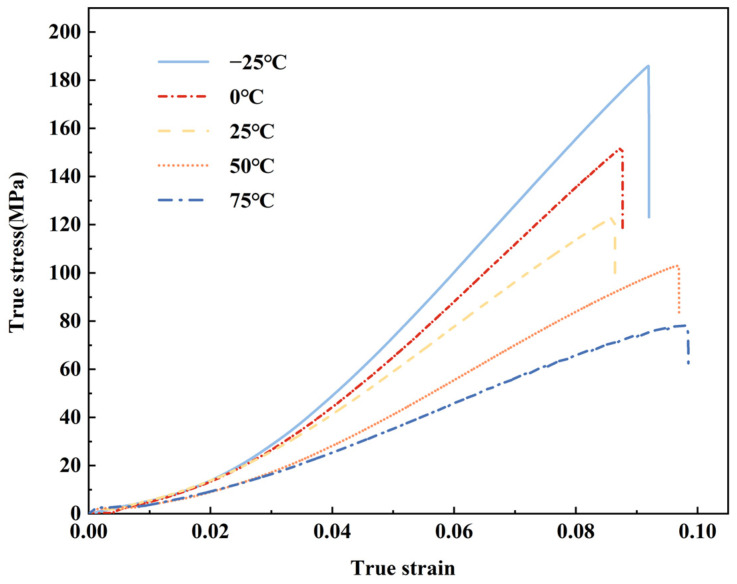
True stress–strain curves for LGFPP at different temperature.

Figure 5 illustrates the real stress–strain curve of glass fiber reinforced polypropylene at various temperatures. Similar to the previous analysis in Section 3.1, this curve exhibits both an ascending and descending section, depicting the entire stretching process. Notably, 25 °C serves as the threshold separating the five temperatures, enabling us to categorize them into low- and high-temperature environments. Comparing the results to those obtained at 25 °C, we observe an increase in maximum strain at higher temperatures and a decrease at lower temperatures. Specifically, the tensile fracture strain rises by 6.7% and 14.6% when the temperature shifts from 25 °C to −25 °C and 75 °C, respectively. This phenomenon arises due to the sluggish molecular movement in low temperature environments and the material’s softening effect in high-temperature environments. These factors hinder the breakage of molecular chains within the material, resulting in an elongated tensile length of the sample. Consequently, the tensile fracture strain is affected and ultimately elevated.

**Figure 6:**
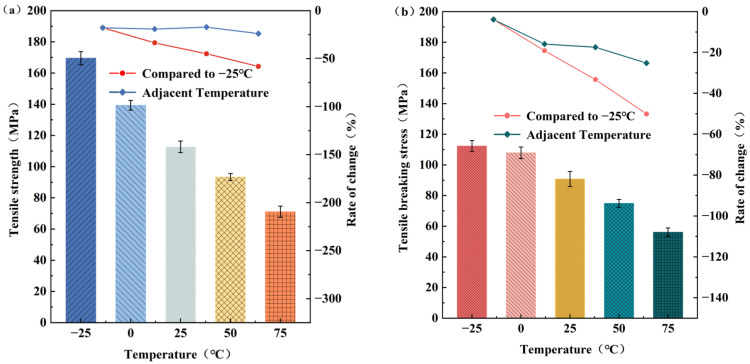
Variation curves of tensile strength and tensile breaking stress of long glass fiber-reinforced polypropylene at different temperatures: (**a**) Diagram of tensile strength variation; (**b**) diagram of tensile breaking stress variation.

Figure 6a demonstrates a decrease in tensile strength as the temperature increases. Compared to −25 °C, the tensile strength exhibits reductions of 17.8%, 33.5%, 44.9%, and 58.1%, respectively. Additionally, Figure 6b illustrates a decrease in tensile fracture stress with increasing temperature. Compared to −25 °C, the tensile fracture stress experiences decreases of 3.9%, 19.2%, 33.3%, and 50.1%, respectively. The rates of reduction in tensile fracture stress between adjacent temperatures are 3.9%, 15.9%, 17.5%, and 25.2%, respectively. Elevated temperatures induce the formation of cracks within the polypropylene matrix, thereby weakening the energy transfer between the long glass fibers and the matrix. Consequently, the required force for specimen pull-off diminishes due to intensified molecular movement, pronounced softening, and compromised energy transfer. Consequently, the experimental results reveal a declining trend in ultimate strength, tensile fracture stress, and tensile fracture strength.

### 3.3. The Effect of Strain Rate Temperature Coupling on LGFPP

**Figure 7:**
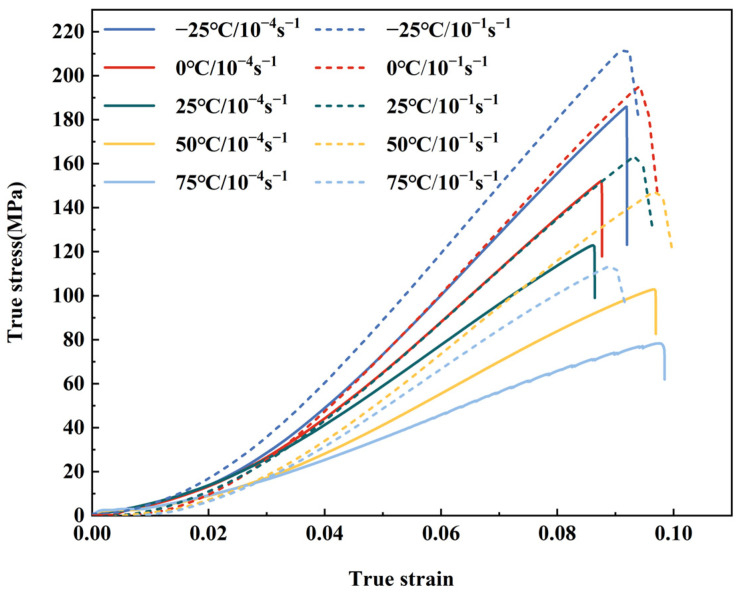
True stress–strain curves for LGFPP at different strain rates and temperature.

**Figure 8:**
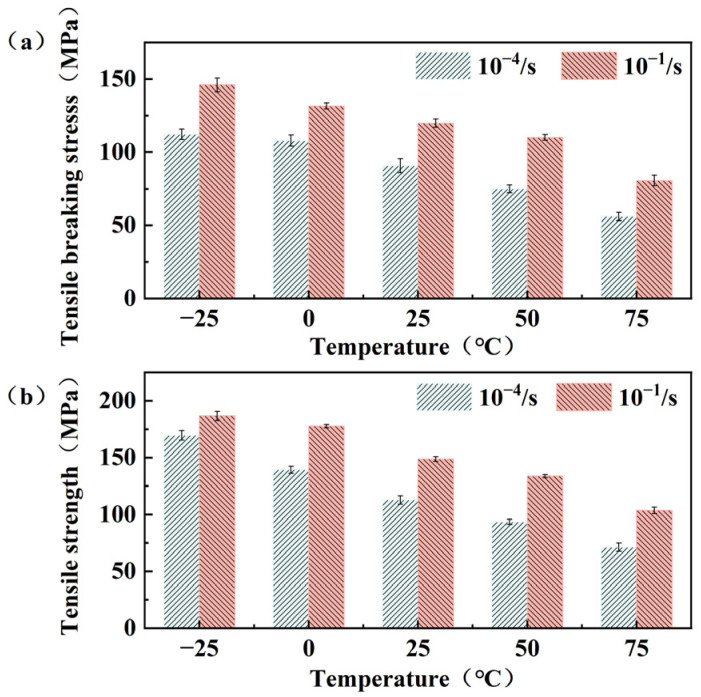
Comparison diagram of tensile strength and tensile fracture stress under strain rate–temperature coupling: (**a**) Diagram of tensile strength variation; (**b**) diagram of tensile breaking stress variation.

**Figure 9:**
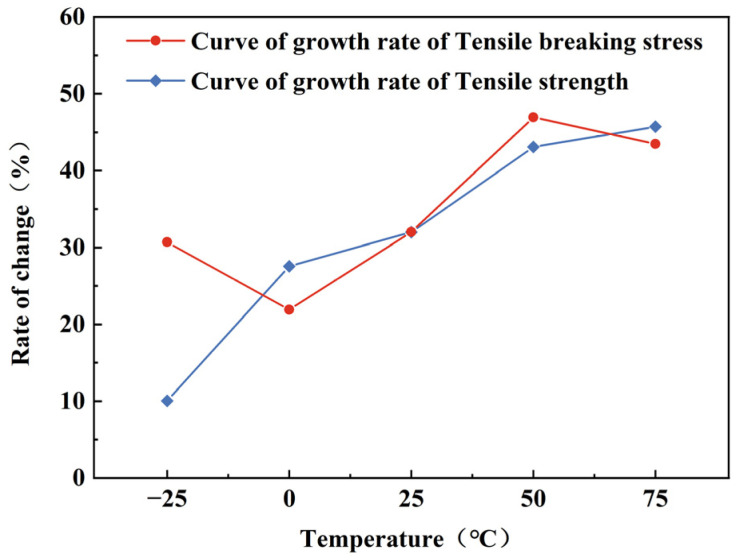
The change curve of tensile strength and tensile fracture stress growth rate at different temperatures with strain rate of 10^−1^ compared to 10^−4^ s^−1^.

In order to comprehensively examine the influence of strain rate–temperature coupling and enhance visual clarity, a series of tensile tests were conducted at five temperatures (−25, 0, 25, 50, and 75 °C) using a higher selected strain rate of 10^−1^ s^−1^. These results were compared with those obtained at a strain rate of 10^−4^ s^−1^, leading to the generation of Figure 7, Figure 8 and Figure 9. This approach facilitates a more thorough understanding and visual representation of the effects under consideration.

Figure 7 presents a comparative analysis of the true stress–strain curves obtained at different temperatures for the two strain rates. It is evident that, within the same strain rate conditions, the ultimate strength, maximum tension force, and fracture force all exhibit a declining trend with increasing temperature. Conversely, under constant temperature conditions, the maximum strain and ultimate strength exhibit an increasing trend with higher strain rates, while the maximum force and fracture force show a decrease. Notably, at 75 °C, the maximum strain appears to decrease as the strain rate increases. This observation can be attributed to the influence of the higher strain rate, which limits the specimen’s ability to distribute the load adequately. Consequently, the matrix and fibers experience rapid failure due to softening phenomena within a relatively short duration.

Figure 8a illustrates the relationship between tensile fracture stress and strain rate at same temperatures. It is evident that the tensile fracture stress increases with higher strain rates. The rate of change in tensile fracture stress between the two strain rates can be observed from the five points represented by the red curve in Figure 9. The curve demonstrates an overall increasing trend, with a decreasing growth rate as the temperature transitions from −25 to 0 °C and from 50 °C to 75 °C. This highlights the substantial influence of low and high temperature on the tensile fracture stress when strain rate is elevated. 

Moving to Figure 8b, it demonstrates that at the same temperature, tensile strength exhibits significant increases at higher strain rates. There is an overall increasing trend in the growth rate of tensile strength between the two strain rates as the temperature rises in Figure 9. Within the temperature range of −25 to 75 °C, the tensile strength for strain rates of 10^−4^ and 10^−1^ s^−1^ decreased by 58.1% and 41.5%, respectively. This indicates that as temperature and strain rate increase, the tensile strength is influenced by the strain rate, and the tendency for a decrease in tensile strength slows down from −25 to 75 °C. The slowing down is attributed to the fact that the tensile strength is affected by the strain rate, an increase in which will not provide enough time for the specimen to deform and thus exhibit greater tensile strength. And the overall decreasing trend of tensile strength is mitigated by the combined effect of temperature.

### 3.4. Microscopic Morphology of Fracture Surface

**Figure 10:**
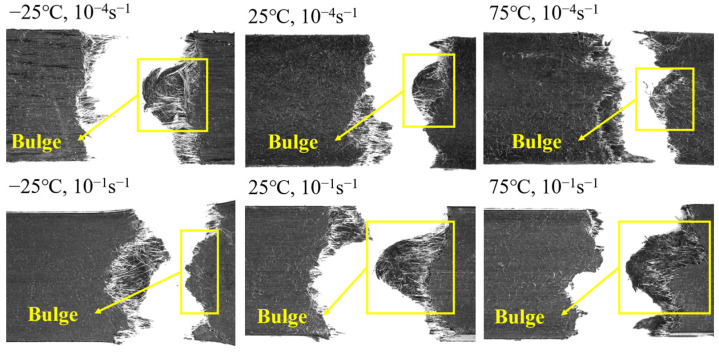
Fracture graph of LGFPP failed samples.

Figure 10 presents the fracture surface of the specimen under different strain rates and temperatures. It can be observed that the fracture surface exhibits a non-flush appearance, with the fibers entangled around a portion of the matrix and pulled out together, resulting in an elevated fracture. This particular phenomenon is not influenced by the strain rate or temperature but rather attributed to the injection molding process [34,35].

**Figure 11:**
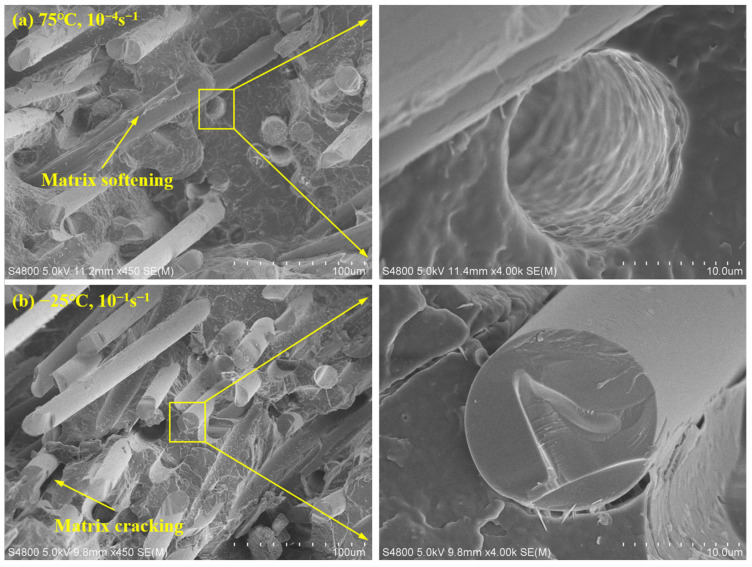
SEM micrographs of LGFPP sample: (**a**) Fiber pull out; (**b**) fiber breakage.

During the tensile process, the failure of the specimen is primarily characterized by two phenomena: fiber breakage and pull-out. Figure 11a displays small round holes within the matrix, which remain after the fibers have been pulled out. Moreover, in the microform at low strain rates, the predominant form of fiber damage is pull-out. The surface of long glass fibers exhibits flocculation, influenced by lower strain rates and higher temperatures. As the glass fibers are pulled out, the polypropylene matrix undergoes softening and elongation during the stretching process. In the case of high strain rates, Figure 11b illustrates the microscopic morphology of the specimen. When the fractured long glass fibers are partially magnified, their uneven surfaces and frequent fractures become evident. This indicates that fiber failure primarily occurs through fracture at high strain rates. The overall smoothness of the glass fiber surface is predominantly attributed to the high strain rate. Additionally, the sample fracture demonstrates characteristics of brittle fracture, and noticeable cracks are observable in the polypropylene matrix under lower temperatures.

**Figure 12:**
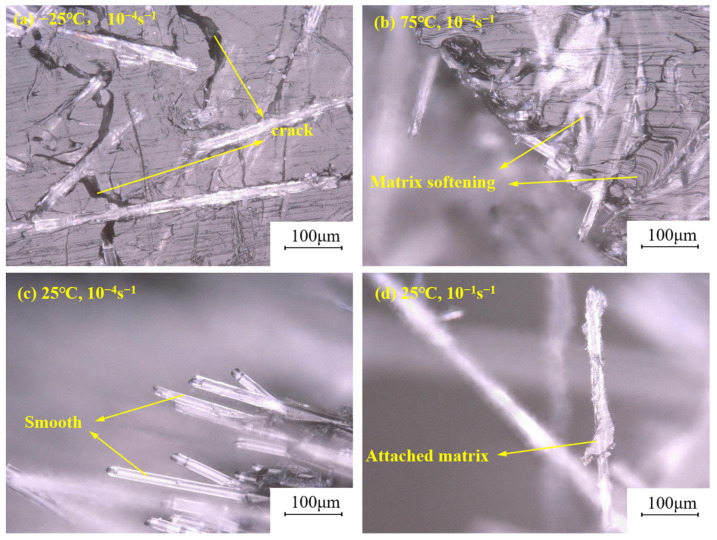
Microscopic figure of LGFPP samples at different loading rates and different temperature.

To visualize the temperature-induced changes within the polypropylene matrix spanning from −25 °C to 75 °C, an ultra-deep field microscope was employed, yielding Figure 12. In Figure 12a,b, particular attention was directed towards examining the external surface of the fractured sample. Evident cracks were observed within the polypropylene matrix at −25 °C, alongside a notable softening phenomenon at 75 °C. Figure 12c,d offer a comparative analysis of long glass fibers at identical temperatures but varying strain rates. Under higher strain rates, the matrix adhered to the fiber experienced fracture, owing to the sample’s propensity to fracture in multiple locations at high strain rates. Consequently, the matrix attached to the surface of the long glass fiber was instantaneously displaced.

## 4. Conclusions

This paper investigates the behavior of long glass fiber-reinforced polypropylene composites under unidirectional tension. Specifically, it examines the impact of different strain rates and temperatures on the mechanical properties, thereby providing insights for practical material applications. The following conclusions are drawn from this study:

(1) The strain rate exerts a significant influence on long glass fiber-reinforced polypropylene composites. As the strain rate increases from 10^−4^ to 10^−1^ s^−1^, the tensile stress, tensile strength, and tensile fracture stress demonstrate an upward trend.
(2) Temperature variations affect the performance of long glass fiber-reinforced polypropylene composites. Notably, when the temperature rises to 75 °C compared to −25 °C, matrix cracks become evident, accompanied by a weakening of energy transfer between the long glass fibers and the substrate. Consequently, the tensile strength and tensile fracture stresses experience an increase.
(3) The coupling of strain rate and temperature yields a combined effect on the tensile strength and tensile fracture stress. At temperatures ranging from −25 °C to 0 °C and from 50 °C to 75 °C, the growth rate of tensile fracture stress is significantly influenced by temperature. The strain rate demonstrates a considerable impact across the temperature range of 0 °C to 50 °C, with an overall increasing trend observed in the growth rate of tensile fracture stress. Additionally, the growth rate of tensile strength exhibits an upward trend when comparing a strain rate of 10^−1^ to 10^−4^ s^−1^.
(4) The fracture morphology of the samples reveals that fiber failure predominantly occurs in the form of long glass fiber fracture and pull-out. Fiber pull-out is more prevalent at lower strain rates, while fiber breakage is observed at higher strain rates. The matrix tends to fracture at lower temperatures and undergoes softening at higher temperatures.

## Figures and Tables

**Table 1:** Material parameters.

Material	Density (g·cm^−3^)	Mass (g·cm^−2^)
Long Glass fiber	2.54	760
Polypropylene	0.91	770

**Table 2:** Test parameter design table.

Experimental Group	Strain Rates (s^−1^)	Temperature (°C)
1	10^−4^	25
	10^−3^	
	10^−2^	
	10^−1^	
2	10^−4^	−25
		0
		25
		50
		75
3	10^−4^/10^−1^	−25
		0
		25
		50
		75

## Data Availability

The data presented in this study are available on request from the corresponding author.
